# L2 epitope presenting virus-like particles as broad-spectrum vaccine candidates targeting genus beta human papillomaviruses

**DOI:** 10.21203/rs.3.rs-7903159/v1

**Published:** 2025-11-23

**Authors:** Huber Bettina, Schüchner Stefan, Shafti-Keramat Saeed, Mohr Katharina, Kirnbauer Reinhard, Ogris Egon

**Affiliations:** Medical University of Vienna; Max Perutz Labs, Vienna Biocenter Campus (VBC); Medical University of Vienna; Max Perutz Labs, Vienna Biocenter Campus (VBC); Medical University of Vienna; Max Perutz Labs, Vienna Biocenter Campus (VBC)

**Keywords:** human papillomavirus (HPV), sequential immunization strategy for mAb generation, chimeric VLP, L2 minor capsid protein, cutaneous genus beta HPV, warts, non-melanoma skin cancer, vaccine

## Abstract

Licensed human papillomavirus (HPV) vaccines do not target cutaneous ßHPV types implicated in skin cancer development in immunosuppressed individuals. The conserved N-terminus of minor capsid protein L2 contains cross-neutralization epitopes, offering the opportunity to develop broad-spectrum vaccine candidates. A sequential immunization strategy using N-terminal L2 fragments of five ßHPVs induced type-common humoral immunity. Four monoclonal antibodies (mAb) were generated that cross-neutralized multiple ßHPV types and conferred in vivo protection against potentially oncogenic HPV5/38/24. MAb epitopes were inserted individually into the DE-surface loop of HPV16 L1 virus-like particles (VLP). Immunizations with chimeric VLPs induced cross-neutralizing antibodies against multiple ßHPV types and conferred in vivo protection against HPV5 infection. Chimeric VLPs displaying ßHPV-L2 cross-neutralizing epitopes are promising broad-spectrum vaccine candidates against the plethora of ßHPVs.

Immunization of patients prior to immunosuppression may reduce the burden of ßHPV infection and thus lower their highly increased risk to develop keratinocytic skin cancer.

## Introduction

Infection with cutaneous genus beta human papillomavirus (ßHPV) types has been implicated in the development of keratinocyte carcinoma or non-melanoma skin cancer (NMSC) development in immunosuppressed patients, acting as adjunct to the main carcinogen ultraviolet (UV) radiation. Patients with the rare genodermatosis Epidermodysplasia verruciformis (EV) show a dramatic increase in the development of cutaneous squamous cell carcinoma (SCC) on sun exposed body areas early in life [1]. Most often, ßHPV5 or 8 have been identified within the tumors and thus been classified as potentially oncogenic [2, 3]. Immunosuppressed patients like organ transplant recipients (OTR) or HIV-positive individuals have an up to 100-fold increased risk to develop NMSC, i.e. SCC and basal cell cancer (BCC) [4]. In these cancers, however, no particular ßHPV type predominates and only very few cancer cells contain ßHPV DNA, contrary to the case with HPV5/8 in EV-SCC [5], or HPV16/18 in cervical cancer (CxCa), in which every cell contains and expresses viral oncogenes. Of note, in non-EV patients ßHPV DNA is more often detected in the pre-cancerous actinic keratosis rather than the SCC itself [6–8]. Consequently, it has been proposed that ßHPV may act through a ‘hit-and-run’ mechanism to prevent apoptosis of UV radiation damaged cells [9, 10]. This results in accumulation of DNA damage and driver mutations leading to progression to cancer. A large body of evidence supports the hypothesis that ßHPVs contribute to human skin cancer formation, and the synergy between ßHPV types and UV exposure in skin carcinogenesis has been shown in transgenic and in natural infection mouse models [11]. Importantly, some ßHPV types can deregulate the immune system, inhibit DNA damage response and target known human tumor suppressors [12–14]. Although Strickley et al. [15] have argued that ßHPV skin colonization drives immunity and may protect against skin carcinogenesis, their using of the mouse papillomavirus MmuPV1 may not be an appropriate comparable model for cutaneous HPVs. A possible role of ßHPV in NMSC development in the immunocompetent population is controversial, since ßHPV are part of the commensal skin microflora and transmission occurs already very early (within days after birth) and throughout the lifespan [16]. Nevertheless, a vaccine that targets cutaneous ßHPV types would be highly desirable for immunosuppressed individuals threatened by their greatly increased risk for skin cancer.

Since no particular ßHPV predominates in NMSC, a broad-spectrum vaccine with cost-efficient reduced complexity would be most beneficial. One approach is based upon the minor structural protein L2 that on its own does not form any structure like L1 capsomers or virus-like particles (VLP), but whose N-terminus contains several conserved cross-neutralization epitopes that induce low-titers of cross-neutralizing antibodies (reviewed in [17]). Unfortunately, L2 is hidden in the virus capsid and does not provide any benefit in vaccinations with co-assembled L1 + L2 VLP [18, 19]. To improve L2’s low immunogenicity, several approaches have been deployed including L2 multimers, fusing L2 peptides to immunostimulatory molecules like thioredoxin, or repetitive display of such epitopes on various platforms like bacteria, bacteriophages or HPV L1 VLP (reviewed in [20, 21]). The latter approach includes RG1-VLP that present the 20 amino acids (aa) long HPV16 L2 cross-neutralization epitope “RG1” (residues 17–36) inserted in the DE surface loop of HPV16 L1 as chimeric fusion protein [22–24]. Immunization with RG1-VLP that display repetitively (360x) and closely spaced the RG1 epitope on the HPV16L1 VLP-surface provides broad cross-protection against all 13 mucosal high risk (hr) types and other pathologically relevant low risk (lr) types in mice [22]. In addition, cross-neutralizing antibodies against a limited number of cutaneous HPV types, including HPV2/3/5/27/76 have been detected as well, but comprehensive protection against the diverse and large group of common cutaneous HPVs would need additional refinement of vaccine antigen.

Cross-neutralization epitopes of L2 have been predominantly identified for HPV16, and experimental vaccines using homologs to RG-1 (aa 17–36), or aa 56–75 [25], or aa 108–120 [26] of other types including mucosal hr HPV45, or cutaneous ßHPV5 and ßHPV17 have been developed eliciting a limited spectrum of protection [27]. In addition, we have previously designed RG1-VLP using a consensus ßHPV ‘RG1’ VLP as a broad-spectrum vaccine candidate to prevent βHPV-associated cutaneous cancers, that provide protection against challenge with βHPV 5, 76 and 96 [28]. However, identification of additional cross-neutralization epitopes could increase the spectrum of protection against cutaneous types and potentially improve L2 antibody titers. It is still unclear, however, which L2 sequences are exposed on native virions and if there are differences between HPV types (reviewed in [17]). In a current model of infection, initial L1 binding to heparan sulfate proteoglycans of the basement membrane causes a conformational change of L1 and cleavage of L2 at the N-terminus by furin, exposing L2 cross-neutralization epitopes [29] and a hypothetic keratinocyte entry receptor binding site. For HPV16 L2 17–36 it was shown that the epitope is increasingly exposed a few hours after initial viral attachment [30]. Thus ideally, an L2-based vaccine would target a sequence readily exposed on the viral capsid during early steps of infection.

Using a strategy of sequential immunizations with L2 protein N-terminal fragments of several different ßHPV types, we generated monoclonal antibodies (mAb) that (cross-) neutralized a panel of ßHPV types *in vitro* and *in vivo*. Peptide arrays were used to map the HPV5 L2 epitopes and chimeric VLP displaying the identified cross-neutralization epitopes on the HPV16 L1-VLP surface were generated. A protective antibody response against experimental pseudovirion (PsV) challenge was induced in immunized mice and rabbits. The results demonstrate the feasibility to generate cross-neutralizing mAbs and L2-targeting chimeric VLP vaccine candidates to protect against the plethora of ßHPV types implicated in keratinocytic skin cancer development.

## Materials and Methods

### Beta L2 proteins

N-terminal fragments of L2 with aa residues 10–142 (10–141 for HPV76, 10–143 for HPV96, 13–130 for HPV16) of HPV5/8/16/20/24/38/76/92/96 (HPV5 M17463; HPV8 M12737; HPV16 K02718; HPV20 U31778; HPV24 U31782; HPV38 U31787; HPV76; HPV92 AF531420 and HPV96 AY382779 Y15174; accessed on pave.niaid.nih.gov [31]) were each inserted into the NcoI-XhoI sites of pET28a(+) (GeneScript USA Inc). Plasmids were transformed into Rosetta2 (DE3) cells (Novagen) and protein expression induced by addition of Isopropyl-1-thio-β-D-galactopyranoside (IPTG). Expression of 6xHis-tagged L2 proteins was verified by Western blot using an anti-His-tag mAb (1:3000; Sigma H1029). Proteins were purified by Ni-NTA chromatography (spin column kit; Qiagen, #31314) under denaturing conditions followed by electro-elution from SDS-polyacrylamid gel in an Elutrap device (Whatman).

### Monoclonal antibody (mAb) generation

Balb/c mice were immunized sequentially with four or five different ßHPV N-terminal L2 protein fragments aa10–142 (10–141 for HPV76) described above (suppl. table 1). For the generation of mAbs 1A1 and 3C6, mice were immunized with 16L1–6B11middle chimeric VLP for the generation of mAbs 1A1 and 3C6. Briefly, mice were injected on days 1, 14, and 35 with a 1:1 emulsion of antigen and adjuvant. A minimum of 21 days after the third injection mice received a final boost w/o adjuvant and splenocytes were fused with X63-Ag8.653 myeloma cells 4 days after the boost. Hybridoma cell clones were established in HAT selective medium and hybridoma supernatants were screened for the presence of specific IgG antibodies 8 days post fusion. Fusion clone screening was done by ELISA using the N-terminal L2 protein fragments of HPV5/8/20/38/76 as antigens, and by furin-cleaved pseudovirion-based neutralization assay (FC-PBNA) (see below) using eight ßHPV types. Positive candidates were monoclonized and re-tested by ELISA and FC-PBNA. Isotypes were determined with the Pierce Rapid Antibody Isotyping Kit – Mouse (Thermo Scientific, 26178) according to the manufactureŕs instructions.

### Epitope mapping

Epitope mapping of clones 7C1, 7G9, 6B11 and 7F6 was performed by ELISA on GST-HPV5 L2 fragment fusion proteins. HPV5 L2 fragments aa10–50, aa36–80, aa73–110 and aa100–142 were cloned into pGEX4T-1 (BamHI-NotI site; GenScript USA Inc.), transformed into Rosetta2 (DE3) cells and protein expression induced by IPTG. Expression was verified by Western Blot using an anti-GST mAb (1:250; GE Healthcare). The fusion proteins were purified by Glutathione Sepharose 4B (GE Healthcare) chromatography according to the company’s instruction, and quantified by SDS-PAGE/Coomassie staining in comparison to lysozyme standards. Epitope mapping with GST-peptides (C-terminal tag) was performed by ELISA using Glutathione-coated 96 well plates (Pierce, #15140) according to the company’s protocol. Subsequent fine epitope mapping was performed by ELISA with synthetic HPV5 L2 15mer peptides with 8aa overlaps using MaxiSorp plates (Nunc; ThermoFisher Scientific, #442404) as well as truncated biotinylated peptides (with a biotin-Ahx tag; GenScript) using Streptavidin plates (Nunc; ThermoFisher Scientific, #436014; see suppl. table 2).

### mAb purification

Hybridoma supernatants were purified over a HiTrap Protein G column using an Äkta liquid chromatography system (Cytiva). Fractions with highest mAb concentrations (A280nm measurements using a NanoDrop; ThermoFisher Scientific) were pooled, concentrated using Amicon Ultra-4 column (Millipore) with a 50 kDa cutoff and desalted using PD Mini Trap G-25 (GE Healthcare) columns according to the companies’ instructions. If not otherwise stated, used mAb concentrations were 2.6 ^mg^/_ml_ for mAb 7C1; 3.6^mg^/_ml_ for mAb 7G9; 5.3 ^mg^/_ml_ for mAb 6B11; 3.8 ^mg^/_ml_ for mAb 7F6; 2.25 ^mg^/_ml_ for mAb 1A1 and 0.56 ^mg^/_ml_ for mAb 3C6. In addition, mAbs 7C1, 7G, 6B11 and 7F6 were cloned, recombinantly expressed (Icosagen AS, Estonia) and re-analyzed in pseudovirion-based neutralization assays (PBNA) in a 1^mg^/_ml_ concentration.

### Western blot with L2 fragments

Ni-NTA agarose purified 6xHis-tagged N-terminal fragments of L2 proteins were separated by 15% SDS-PAGE, transferred to nitrocellulose, and detected with either anti-His tag antibody (1:4000, Genscript, A00186) or with the purified mAbs generated in this study (at 1μg/ml). Western blot signals of three technical replicates were quantified with ImageQuant software, adjusted to loaded amounts as determined with anti-6xHis tag antibody, and are shown on a logarithmic scale relative to HPV5 L2 (set to 1000).

### Cloning and expression of peptide oligomers

N-terminal 6x His-tagged peptide oligomers were generated by DNA-synthesizing and expressing the four identified HPV5 L2 epitopes 7C1 (aa25–31), 7G9 (aa68–75), 6B11 (aa85–95) and 7F6 (aa123–136) individually as pentameric repeats using the bacterial pET28a expression vector (BamHI-XhoI site; GenScript USA Inc.). Similarly, an octamer consisting of four tandem repeats of each epitope was generated as well (termed beta epitopes x2; βEx2) (suppl. Figure 3). Protein expression was induced by IPTG after transformation into Rosetta2 (DE3) cells (Novagen) and expression was verified by SDS-PAGE/Coomassie staining and Western blot using the respective L2 mAbs. Proteins were purified using Ni-NTA spin column (Qiagen). The 7G9×5 polymer was additionally cloned into pVitro1-neo-mcs (BamHIAvrII site; with a C-terminal 3xGS linker and 6xHis tag) and expressed in 293TT cells as described below for VLP and PsV productions.

### Design of chimeric VLP

The HPV16 L1 backbone was based upon the European variant 114K L1 (accession no. EU118173) and synthesized in a codon optimized version for mammalian expression (GenScript). A pVitro1-neo-mcs library approach was employed, in which Pfl23II and MreI restriction enzyme (RE) sites were introduced close to the HPV16L1 aa136/137 (DE-loop) insertion site to facilitate cloning of oligonucleotides that encode the respective HPV5 L2 cross-neutralization epitopes flanked by GSGS linkers for expression as L1-L2 fusion proteins. Oligonucleotides encoding the following HPV5 L2 epitopes were cloned into the DE-loop of HPV16L1: 7C1mod (aa24–34), 7G9mod (aa60–72), 6B11short (aa84–98), 6B11middle (aa7998), 6B11long (aa66–98) and 7F6mod (aa127–138) (all synthesized by GenScript USA Inc.; suppl. table 6). Single stranded complementary oligos were annealed with sticky RE site overhangs, 5’-phosphorylated, ligated into the Pfl23II plus MreI digested and de-phosphorylated HPV16L1-pVitro vector and transformed into Ag1 competent cells (Agilent). Colonies were screened by PCR by negative (forward L1 primer 5’- GCAGTGGGACACCC-3´ and an HPV5 L2 unrelated reverse primer 5’- CGCCATTGCAAGGTG-3´) and positive selection (L1 library primers; forward 5’- GAATCTCCGGACACCC-3´ and reverse 5’-CACAGCTGGGTCTGC-3´), and sequenced (Eurofins Genomics) using the L1 library primers. Plasmids were purified using Qiagen’s Maxi or Mini Plasmid Kits.

### Expression, purification and characterization of VLP

Chimeric VLP were produced in human 293TT cells according to published protocols [32, 33]. Briefly, 13–16*10^6^ 293TT cells were plated into 175cm2 flasks and the following day transfected with 38μg L1-beta L2mod plasmid DNA (Turbofect, ThermoFisher Scientific, #0531). 48 hours after transfection cells were lysed in PBS + 9.5mM MgCl_2_ + 0.5% v/v Triton-X100 + 0.25% w/v Ammonium sulfate + 0,1% Benzonase (of a 25U/μl stock; Merck). For particle maturation, cell lysates were incubated 20–24 hours at 37°C before VLP were salt extracted and purified by ultracentrifugation on Optiprep step gradients (27%, 33%, 39%; Sigma D1556).

Viral capsid fusion proteins were identified by SDS-PAGE/Coomassie staining, or western blot using mAb Camvir-1 (1:10,000 dilution; BD Pharmingen) [34] directed against a linear epitope of HPV16L1, and the respective beta L2 mAbs. Self-assembly of chimeric proteins was examined by transmission electron microscopy (TEM) using a JEOL 1010 electron microscope at 80kV. Briefly, purified VLP were adsorbed onto glow-discharged copper grids, fixed with 2.5% glutaraldehyde and negatively stained with 1% uranylacetate. Micrographs were taken at 30,000x magnification.

### Immunizations

Groups of 6–8-week-old Balb/c mice (n = 5 per group; obtained from Charles River, Germany) were immunized subcutaneously (s.c.) with respective Optiprep-gradient purified chimeric VLP (2.5 μg/dose), or pentamers (10μg/dose), in a 3-dose regimen at weeks 0-2-4 using complete (prime) or incomplete (boosts) Freund’s adjuvant (CFA, IFA; Sigma Aldrich). Pre-bleeds and final bleeds were obtained at weeks 0 and 6 and pooled for the respective groups.

New Zealand White (NZW) rabbits were immunized (n = 1 per antigen) at Charles River (France) using 20μg of VLP adjuvanted with 200μg of Alum (2% Alhydrogel; Invivogen, #vac-alu-250) in a 3-dose regimen at weeks 0-3-6. Pre-bleeds and final bleeds were taken at weeks 0 and 8, respectively.

### ELISA

Sandwich ELISA: Antigenicity of chimeric VLP as compared to wild type (wt) HPV16L1 VLP was analyzed by ELISA. VLP preparations were quantified by SDS-PAGE/Coomassie staining by comparing the intensity of the L1 band to serial dilutions of a bovine serum albumin (BSA) standard. In short, Maxisorp 96-well plates (Nunc) were coated with 0.3μg native VLP in 100μl PBS per well overnight at 4°C. For denaturing conditions, 0.3μg VLP in 100μl 0.2M NaHCO_3_ (pH 10.6) + 0.01M freshly added dithiothreitol (DTT) per well were plated onto the ELISA plate without a lid overnight at 37°C. On the next day, wells were washed with PBS, blocked with 0.5% milk/PBS, and serial dilutions of antibodies (all ranging from 1:200–1:800) added in triplicates for one hour at room temperature (RT). Antibodies used were neutralizing anti-HPV16 L1 mouse mAb H16.V5, non-neutralizing mAb Camvir-1, or the respective beta L2 mAbs. After a PBS wash step, secondary antibody goat anti-mouse or goat anti-rabbit IgG-HRP (Bio-Rad, #STAR134P) was added at 1:10,000 dilution for 1h at RT, followed by substrate 2,2’-azino-bis(3-ethylbenzothiazoline-6-sulphonic acid) (Roche). The OD at 405nm was determined with an ELISA reader (Opsys MR, Dynex Technologies) and positive titers are reported for OD values greater than the mean OD of VLP without 1st antibody plus 3 standard deviations (SD).

L2 peptide ELISA: Biotinylated or GST-labeled, overlapping or truncated peptides of HPV5 L2, or the respective HPV5 L2 epitopes were synthesized by GenScript. For epitope mapping with biotinylated peptides, 1 μg biotinylated peptide in 100μl coating buffer (1M Tris/HCl (pH7.4) + 1M NaCl + 0.001% Tween-20) per well was plated at 4°C overnight onto Streptavidin plates (Nunc immobilizer Streptavidin F96 clear). For epitope mapping using GST-L2 peptides, glutathione (GSH)-coated plates (ThermoFisher Scientific; 15140) were washed with PBS and wells were coated with 1^μg^/_ml_ GST-proteins in PBS at 4°C overnight. After peptide binding, wells were washed with PBST for 5 minutes, blocked with 1% milk/PBS for one hour at RT, washed again and incubated in triplicates with serial dilutions of the respective mAb or antisera (1:200–1:204,800) for one hour at RT on an ELISA plate shaker. HRP-coupled secondary antibody and substrate were added as above. The OD at 405nm was determined and titers are reported for values greater than the mean OD of pre-immune sera plus 3 standard deviations (SD).

### HPV Pseudovirion Production

Pseudovirions were produced in 293TT cells according to published protocol using 19μg HPV L1/L2 expression vector, 19μg reporter plasmid (pCLucf for firefly luciferase or pYSEAP for secreted embryonic alkaline phosphatase) and Turbofect (ThermoFisher Scientific) [32, 33]. Plasmids for L1 and L2 of HPV16 and MmuPV-1 were kindly provided by J. Schiller, NCI; for HPV5 L1 and L2 by C. Buck, NCI; for HPV76 L1 and L2 by J. Dillner, Karolinska Institute; and for MnPV L1 and L2 by D. Hasche/F. Rösl, DKFZ.

Furin pre-cleaved PsV (fcPsV) containing the luciferase reporter were generated in 293TTF cells following a modified standard protocol [35]. Maturation was carried out for 48 hours to achieve a more efficient L2 cleavage of particles.

### Pseudovirion-based neutralization assays

The L1-based PBNA assay was performed as previously described [32]. Briefly, 3*10^4^ 293TT ^cells^/_well_ were plated onto a 96-well plate and infected in duplicates with SEAP-containing PsV pre-incubated without or with respective mAb or mouse sera for one hour. Infection was assessed three days later by measuring the SEAP signal at 405nm (Opsys MR, Dynex Technologies) after addition of 4-nitrophenyl phosphate disodium salt hexahydrate (Sigma; N2765) dissolved in diethanolamine (Sigma). Serum dilutions showing at least a 50% reduction in SEAP signal compared to pre-serum were considered neutralizing.

The L2-based PBNA were performed according to Day et al. [36] or Wang et al. [35]. For the first assay, 2*10^6^ HaCaT cells were plated onto 96-well plates for 24 hours at 37°C, cells were washed with PBS twice and lysed using PBS + 0.5% Triton X-100 + 20mM NH_4_OH for 5 minutes at 37°C. Following lysis, 100μl PBS was added and removed again for three times to gently wash the remaining extracellular matrix, followed by addition of PsV in 120^μl^/_well_ CHOΔfurin conditioned medium containing 5μg/ml heparin (Gilvasan Pharma). The ECM/PsV mix was incubated overnight at 37°C and then washed with PBS on the next day to remove non-attached PsV. Antibody serial dilutions were added and incubated at 37°C for 1 hour (in triplicates). After this incubation step, 8*10^3^ PGSA-745 cells were directly added to the wells (50^μl^/_well_) and incubated at 37° for further two days. For luciferase encoding PsV, assays were evaluated using the Luciferase Assay System (Promega #E1501) and the Cell Culture Lysis Reagent (Promega #E153A) according company’s instructions in 96-well opaque Optiplates (Nunc). Luciferase activity was measured using the Fluoroskan Ascent FL (ThermoFisher Scientific) plate reader, with 10 second reading time per well. Serum dilutions showing at least a 50% reduction in Luciferase signal compared to an unrelated mAb or pre-serum were considered to be neutralizing. To produce CHOΔfurin conditioned medium, 1*10^6^ cells were grown in 17ml media and supernatants harvested after incubation at 37°C for 4 days.

To perform neutralization assays using fcPsV (FC-PBNA) [35], fcPsV were pre-incubated with or without hybridoma supernatant, or serial dilutions of mAb or (pre-) immune sera (2h at 37°C; in triplicates), added onto pre-plated LovoT cells (3*10^4^/well) and incubated at 37°C for three days. Assays were evaluated with Promega’s Luciferase Assay system as described above.

### Cell lines

293TT cells were cultured in Dulbecco’s Modified Eagle Medium (DMEM) supplemented with heatinactivated 10% fetal calf serum (FCS; BioWest), 1x non-essential aa (NEAA; Gibco) and 400^μg^/_ml_ Hygromycin B (Invitrogen). HaCaT cells were cultivated in DMEM + 10% FCS + 1x Pen-Strep (Gibco). 29TTF and LovoT cells (kindly provided by R. Roden, Johns Hopkins University, USA) were cultivated in DMEM + 10% FCS + 1x NEAA + 1x Pen-Strep + 1x sodium pyruvate (Gibco) + 1x Glutamax (Gibco) and 2^μg^/_ml_ Puromycin dihydrochloride (for 293TTF; Gibco) or 200^μg^/_ml_ Hygromycin B (for LovoT cells). The PGSA-745 and CHOΔfurin cells (a kind gift by Patricia Day, NCI) were cultivated in DMEM + 10%FCS + 1x Pen-Strep + 10mM proline (Sigma).

### In vivo vaginal challenges

The passive immune serum transfer and challenge experiments were performed according to Roberts et al. [37] with six to eight weeks-old female Balb/c mice (Charles River Laboratory; Germany). Briefly, groups of mice received 3mg progesterone sub-cutaneously (Depocon; Pfizer) three days prior to passive immunization (50μg of indicated mAb; intra-peritoneal) with HPV-unrelated mAb/serum (n = 10), respective beta L2 mAbs (n = 5), or a mAb/serum (50μg mAb or 20μl serum) against the respective HPV type (n = 5; mAb H5.A6 (kindly provided by Neil Christensen, Pennsylvania State University, USA) raised against HPV5 L1-VLP; anti-PsV24 or anti–PsV38 as HPV24- or 38-specific antiserum). After 24 hours, 20μl of a PsV-carboxymethylcellulose (CMC, Sigma) mixture (PsV-CMC ratios: 2:1 for HPV5; ~6:1 for HPV24; and 5:1 for HPV38) were installed intravaginally using a positive displacement pipette (Gilson). The cervical mucosa was gently disrupted by 20x rotating a cytobrush (Cooper Surgical Company; C0012) and further 20μl PsV-CMC were applied intravaginally. Three days later, infection was evaluated by intravaginal installment of 20μl luciferin (7.5^mg^/_ml_; Promega) and imaging (10-minute exposure) by the IVIS (Caliper Life Science) or LagoX system (Spectral Instruments Imaging). Data were analyzed by the Living image (IVIS) or Aura Imaging software (LagoX) by drawing uniform regions of interest (ROI) around the luminescence emitting genital area of each mouse to determine the average radiance within the ROI, as well as determine background radiance at an uninfected site of the same mouse. Luciferase activity was measured as p/s/cm2/sr (average radiance). Bioluminescence measurements were performed at the Department of biomedical imaging and image-guided therapy, Division of Molecular and Structural Preclinical Imaging (PIL/EXPNUC), Medical University of Vienna.

For active immunizations with chimeric VLP followed by HPV5 PsV (PsV5) vaginal challenge, 6–8-week-old mice were immunized three times (week 0/2/4) with 2.5μg chimeric VLP, HPV16 L1-VLP, PsV5 (each CFA/IFA adjuvanted), or PBS prior to challenge with PsV5 (2:1 PsV-CMC) as mentioned above.

### Animal welfare

The maintenance of mice and all experimental procedures involved in the generation of mAbs, clones 7C1, 7G9, 6B11, 7F6, 1A1 and 3C6 were conducted according to the Austrian Animal Experiments Act and approved by the Austrian Federal Ministry of Science and Research (BMWFW-66.009/0211-WF/V/3b/2015 and 2020 − 0.040.298) and the animal experiments ethics committee of the Medical University of Vienna. Animal studies (immunizations with polymers and VLPs, and *in vivo* vaginal challenges) were approved by the ethics committee of the Medical University of Vienna and the Austrian Federal Ministry of Science and Research (BMBWF 2020 − 0.594.252) and animal care was in accordance with the guidelines of the Institute for Biomedical Research Vienna. Vaginal challenges were performed under Ketamine hydrochloride/Xylazine anesthesia to minimize suffering. Animals were sacrificed by cervical dislocation.

### Statistics

*In vivo* experiments were analyzed by measuring luciferase activity as p/s/cm2/sr (average radiance) and quantified after background subtraction (measurement at an uninfected site on the same mouse) using Living Image Software (PerkinElmer) or Aura Imaging software (LagoX). Statistical analysis was performed using Graph Pad Prism to evaluate p-values between indicated groups (unpaired, two-tailed t-test, and Welch’s corrected if groups with unequal sizes were analyzed). Results are provided in a descriptive manner with mean and standard deviation. ELISA titers are reported for OD values greater than the mean OD of pre-immune sera plus 3 standard deviations (SD), while PBNA neutralization titers are indicated for reciprocal serum/mAb dilutions resulting in 50% reduction in reporter signal compared to an unrelated mAb or PsV only.

## Results

**Figure 1:**
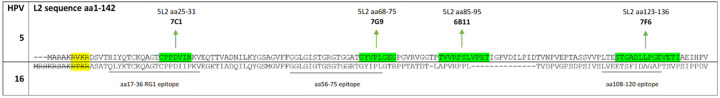
Epitopes of mAbs. Epitopes of cross-neutralizing βHPV5 L2 mAbs are indicated in green on the HPV5 L2 N-terminal protein sequence (upper panel). The N-terminal protein sequence of HPV16 L2 is aligned (lower panel) with known (homologous) HPV16 L2 cross-neutralization epitopes indicated (underlined). mAb 7C1 (HPV5L2 aa25–31) recognized parts of the HPV16 RG1 homolog [24]. MAb 7G9 recognized HPV5L2 aa68–75. This epitope overlaps with the C-terminus of HPV16 L2 aa56–75 cross-neutralization epitope [25]. MAb 7F6 recognized HPV5L2 aa123–136. This cross-neutralization epitope is partially overlapping with a described cross-neutralization epitope of HPV16 L2 aa108–120 [26] but showing a rather dissimilar sequence homology. Mab 6B11 recognized the novel epitope HPV5L2 aa85–95. The (presumptive) furin cleavage sites of HPV5 L2 (aa6-9) and HPV16 L2 (aa9-12) are indicated in yellow. The furin cleavage site was not included in the L2 fragments used for mAb development (crossed out sequence).

The HPV L2 N-terminus is an attractive candidate for broadly cross-neutralizing vaccines because of its higher degree of conservation. We therefore set out to identify cross-protective epitopes in cutaneous βHPV types by generating a panel of mAb, identifying their respective epitopes and evaluating their cross-neutralizing potential. To selectively enrich for broadly cross-reactive antibodies, mice were sequentially immunized in different orders with N-terminal L2 protein fragments of five βHPV types HPV5/8/20/38/76 (aa 10–142, or 10–141 for HPV76, excluding the conserved furin cleavage site (L2 aa 6–9; RTKR [29])). We previously employed such an immunization strategy for the generation of Remazoldye cross-reactive antibodies to visualize multi-coloured molecular weight markers in western blot analysis [38], and similar approaches have been described for HIV-1 or influenza virus [39–41]. Hybridoma supernatants were screened by ELISA using the N-terminal L2 proteins of the five ßHPV types, and subsequently tested for cross-neutralization by FC-PBNA against the same types. Four cross-reactive and cross-neutralizing mAbs were generated from 4 mice, each immunized with an individual sequential immunization protocol (suppl. table 1). Their respective epitopes were mapped first by ELISA using a panel of overlapping HPV5 L2 fragments corresponding to aa10–50, aa36–80, aa73–110, or aa100–142, respectively, that were expressed as GST-fusion proteins (suppl. table 2). Epitope fine mapping was subsequently performed by ELISA on a panel of synthetic 15 aa long, 8 aa overlapping HPV5 L2 peptides spanning the entire aa10–142 sequence, and finally on an additional panel of stepwise truncated peptides. Three of the mAbs bound to epitopes that were either part of, or partially overlapping with homologs of known L2 neutralization epitopes from HPV16 L2 (Fig. 1). Clone 7C1 recognized HPV5 L2 peptide aa25–31, which is homologous to the C-terminal part of the RG1 epitope [24]; mAb 7G9 recognized HPV5 L2 aa68–75, partially overlapping with the far C-terminal part of the HPV16L2 aa56–75 epitope [25]; mAb 7F6 recognized HPV5L2 aa123–136, partially overlapping the HPV16L2 aa108–120 epitope although the aa sequence is considerably different [26]. Clone 6B11, however, recognized an epitope ranging from aa85–95 that represents a novel L2 cross-neutralization epitope. All mAbs were determined to be IgG1 κ light chain isotype.

All mAbs isolated by reactivity to HPV5 L2 were also cross-reactive to L2s of additional βHPV types as determined by western blot using the purified N-terminal L2 fragments of 8 different bHPV types including HPV24, HPV92 and HPV96 that had not been included in the immunizing scheme for the generation of these antibodies (suppl. Figure 1). Reactivity appeared restricted to genus βHPV L2, as mucosal genus αHPV16 L2 was not recognized. Protein G-purified mAbs were analyzed by FC-PBNA for their cross-neutralization spectrum to additional ßHPV and two animal papillomavirus types. Each of them (cross-)neutralized several if not all 8 tested ßHPV PsV with titers ranging from 100- ≥128.000; Table 1), including HPV24, 92 and 96 that were not used for immunization thus indicating cross-neutralization. Despite of the difference in target protein presentation between a western blot and a PBNA, the cross-reactivity results obtained by the two methods were consistent for most mAbs with a few exceptions: mAb 6B11 detected all bHPV type L2 fragments by western blot but showed only low titers in the PBNA against HPV76 and 96, and, likewise, mAb 7F6 strongly detected all L2s by western blot but failed to neutralize HPV76. MAb 7C1 additionally cross-neutralized PsV of animal types Mastomys natalensis (MnPV) and Mus musculus papillomavirus (MmuPV). Additionally, the four mAbs were cloned, recombinantly expressed (Icosagen AS, Estonia) and re-tested in equal concentrations (1^mg^/_ml_) by FC- and L2-PBNA (suppl. table 3). The cross-neutralization profiles were similar to the parental mAbs (Table 1), with the exception that the hybridoma-produced mAb 7F6 did not cross-neutralize HPV96, but the recombinant 7F6 did at low levels.

Although mAbs 1A1 and 3C6 were generated against an extended 6B11 epitope (79–98) displayed by chimeric HPV16L1 VLP (6B11middle; suppl table 6), they exhibited different (cross-)neutralization spectra than mAb 6B11, which (cross-)neutralized all tested βHPV types, while mAb 1A1 (cross-)neutralized 5 of 8 tested βHPV, and mAb 3C6 (cross-)neutralized 7 of 8 tested βHPV, but at much higher titers than 6B11.

**Figure 2:**
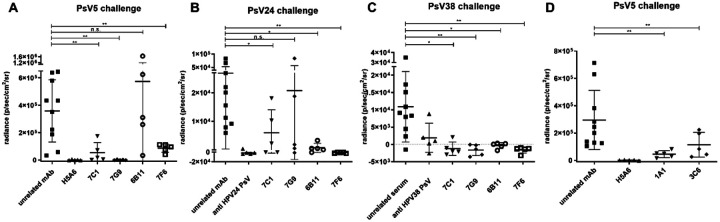
Mabs protect against experimental genital challenge *in vivo*. Progesterone synchronized Balb/c mice (n=5) were passively immunized with 50μg of the respective ßHPV mAb, an HPV-unrelated mAb/antiserum, or an HPV5 type-specific control mAb (mAb H5A6), or 20μl of a polyclonal rabbit antiserum raised against HPV24 or HPV38 PsV prior to experimental challenge at the vaginal mucosa using PsV of HPV5 (A and B), HPV24 (C), HPV38 (D). After 3 days luciferase activity was measured as p/s/cm2/sr (average radiance) and quantified after background subtraction. Shown are statistical differences comparing unrelated mAb/serum to mAbs. The comparison unrelated mAb/serum to the type-specific control was always significant. * p<0.05; ** p<0.005; *** p<0.0005; p>0.05 was considered not significant (n.s.).

*In vivo* protective efficacy of mAbs was tested in the murine vaginal challenge model [37] against βHPV5 and 38, or HPV24 whose L2 had not been used for immunizations in the generation of these mAbs. MAb 7F6 protected against infection with all three PsV types and in two of three cases (HPV24 and HPV38) even to levels of the type-specific control (Fig. 2A, B and C). MAb 7C1 protected against challenge with HPV5 and 38 but less well against HPV24. MAb 7G9 showed protection against HPV5 and 38 and a trend towards protection against HPV24, with 3 of 5 mice showing clear protection.

Finally, mAb 6B11 protected against HPV24 and 38, but not HPV5, although HPV5 had been used twice in its immunization schedule (Fig. 2A and suppl. table 1). However, passive transfer of recombinantly expressed mAb 6B11-Ico showed a trend towards some level of protection against challenge with HPV5 (indicated by the significant differences to both the unrelated mAb and the type-specific control; suppl. Figure 2) which was consistent with the low titers in the cross-neutralization assays against this type (suppl. table 3). Transfer of the other recombinantly expressed mAbs confirmed their protective ability (suppl. Figure 2). Moreover, mAbs 1A1 and 3C6 that were raised against the extended 6B11middle epitope, showed both *in vivo* protection against HPV5 (Fig. 2D), probably reflecting the much higher titers seen in the cross-neutralization assays.

### Oligomeric epitope display induces cross-neutralizing immunity

To determine if the identified cross-neutralization epitopes had the capacity to induce cross-neutralizing antisera, we first used an oligomeric linear epitope display (suppl. Figures 3 and 4) for immunizations (with the exception of 7G9 oligomer (aa68–75) that could not be expressed). Sera raised against the epitope polymers cross-neutralized the homologous HPV types as the parental mAbs with few exceptions, but with considerably lower titers (suppl. table 4 and 5). Thus, while the epitopes per se appeared promising in inducing a broadly cross-reactive immune response, immunogenicity was unsatisfying.

### Chimeric VLP generation and characterization

To increase immunogenicity of the identified novel βHPV L2 cross-neutralization epitopes, individual peptides were genetically inserted into the DE surface loop of HPV16 L1 (between aa residues #136/137) as previously reported [23] with the aim to generate chimeric VLP candidate vaccines particularly targeting the large spectrum of cutaneous βHPV inducing broader protective efficacy than previously generated chimeric VLPs targeting β and αHPV [27, 28]. The inserted L2 epitope sequences were modified to particularly include conserved aa around and within the respective L2 epitopes, and flanked N- and C-terminally by flexible GSGS linkers (Fig. 1 and suppl. table 6). Additionally, since the epitopes of mAbs 7G9 and 6B11 are adjacent to each other in L2, we also inserted a longer sequence (6B11long; aa66–98) comprising both epitopes. Altogether, six fusion proteins were designed, with three particularly focusing on the 6B11 epitope (aa85–95). This included an extended 6B11 epitope, 6B11middle (HPV5 L2 aa79–98), inserted into HPV16 L1 (pos 135/136), which was used for further mAb generation specifically to the 6B11 region. Immunizations with chimeric HPV16L1–6B11middle VLP gave rise to two additional mAbs 1A1 and 3C6.

Chimeric VLPs were generated as described in materials and methods and expression verified by SDS-PAGE/Coomassie staining and western blot (suppl. Figures 5 and 6). TEM confirmed that all six fusion proteins assembled into spherical particles of around 55nm diameter (suppl. Figure 7). For HPV16L1–6B11long, VLP were only sparsely observed (indicated by yellow arrows) reflecting the rather low expression level (suppl. Figure 5). To evaluate antigenicity profile indicating assembled particles, VLP preparations were probed with conformation-dependent HPV16-neutralizing mAb H16.V5, or linear non-neutralizing mAb Camvir-1 by ELISA (suppl. Figure 8). Under native conditions, all six VLP preparations showed stronger reactivity with H16.V5 verifying the presence of conformational neutralization epitope(s), while mAb Camvir-1 reactivity was stronger under denaturing conditions as expected (suppl. Figure 8A-F). Since Camvir-1 recognizes a linear HPV16 L1 epitope also exposed on incompletely assembled particles, some reactivity was also detected under native conditions. Of note, ßHPV L2 mAbs reacted with their respective chimeric VLP under both native and denaturing conditions, but not with HPV16 wt L1-VLP (suppl. Figure 8G), indicating successful presentation of the L2 peptide epitopes by the fusion proteins.

### Immune response to chimeric VLP

To analyze the (cross-)neutralizing immune response induced by chimeric VLP vaccine candidates, groups of mice (four for HPV16L1–6B11middle (not shown) and five for the other chimeric VLP) were immunized three times with a two-week interval. Two weeks after the second boost, immune sera were drawn, pooled for each chimeric VLP and analyzed by L1-PBNA and L2-based FC-PBNA (Table 2). All pooled sera showed high HPV16-neutralizing titers (ranging from 3.200 to 51.200) by L1-PBNA (not tested for antiserum to 16L1–6B11middle) (Table 2, first row). Importantly, immune sera derived from immunizations with chimeric 16L1–7C1mod, 16L1–7G9mod, 16L1–7F6mod and 16L1–6B11long VLP (cross-)neutralized all 8 tested βHPV types (titers ranging from 50 - >3.200), while antisera to 16L1–6B11short (cross-)neutralized 5 of 8 tested types. Individual mouse immune sera to 16L1–6B11middle were tested for neutralization of HPV5 and 20 in order to choose those mice with the highest cross-neutralizing titers for the generation of mAb with improved cross-neutralization titers (not shown). Two additional mAbs 1A1 and 3C6 were generated, which possessed up to 1000-fold increased cross-neutralization titers compared to mAb 6B11, but with a reduced cross-neutralization spectrum (Table 1).

### In vivo protective efficacy

**Figure 3:**
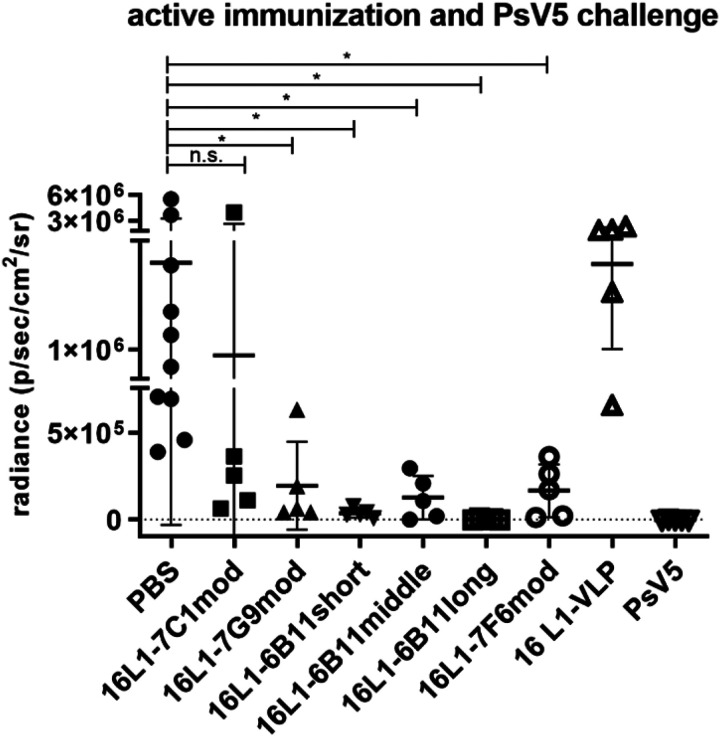
In vivo protective vaccine efficacy against experimental HPV5 PsV challenge. Balb/c mice were actively immunized with indicated chimeric HPV16L1 VLP, parental HPV16 L1 VLP, or HPV5 PsV (2.5μg at weeks 0/2/4 using Freund’s adjuvant), respectively. Mice were challenged vaginally two weeks after the final boost and infection assessed after three days using the IVIS bioluminescence imager. Luciferase activity was measured as p/s/cm2/sr (average radiance) and quantified after background subtraction (measurement at an uninfected site of the same mouse) using Living Image Software (PerkinElmer). Reported are p-values of unpaired, two-tailed t-test, Welch’s corrected if groups with unequal sizes were analyzed; p 0.05, *; p>0.05 were considered not significant (n.s.).

*In vivo* efficacy of vaccine candidates was investigated by a murine vaginal challenge model by active immunization prior to PsV challenge [37]. Groups of mice were immunized three times with one of the six chimeric VLP, respectively, or HPV16 L1-VLP, PsV5, or PBS as controls, followed by intravaginal challenge with PsV5 two weeks after the final boost. Results indicate, that active immunizations with all six chimeric VLPs provided protection against HPV5 challenge (Fig. 3). Complete protection to levels similar to the type-specific group was seen in HPV16L1–6B11long, and HPV16L1–6B11short immunized mice. HPV16L1–6B11middle, −7G9mod and − 7F6mod showed significant differences to the PBS mock control as well as the type-specific PsV5 group indicating partial protection. Some level of partial protection was also seen with HPV16L1–7C1mod that showed no significant difference to both the PBS (p = 0.4) and the type-specific control (p = 0.2).

## Discussion

Licensed mucosal HPV vaccines are based on L1-VLP and protection depends on the types included in the vaccine, as L1-targeted immunity is mainly type-restricted. Inclusion of additional VLP of clinically relevant mucosal and cutaneous HPV types into current (up to nonavalent) vaccines is impeded by technical and economic hurdles.

To address this unmet medical need, we have previously generated chimeric HPV16 L1 VLP that display the broad cross-neutralization epitope ‘RG1’ of the HPV16 L2 minor capsid protein. Vaccination with these RG1-VLP induces broadly cross-neutralizing antisera and provides cross-protection against all mucosal hr and multiple lr HPV types [22–24], and a US NCI-sponsored phase I study in women has been initiated (NCT05985681). Vaccination of small animals with HPV16 RG1-VLP however, has induced cross-neutralization to only a limited number of *cutaneous* types including HPV5, 76, 2, 27, 3 [22, 28]. Further attempts to generate a broad spectrum ßHPV vaccine candidate used the ßHPV17 RG1 homolog inserted into HPV5 L1. Although cross-protection was seen against several tested ßHPV, L1 type-specific protection against HPV5, due to the epitope insertion, was ultimately disrupted [27].

Another study took advantage of the thermostable thioredoxin and an oligomerization domain as a scaffold to display an L2 aa20–38 (the RG1 homolog) multimer generated from 6 to up to 14 different hard to target cutaneous alpha, beta, gamma, nu and mu HPV types [21]. Their leading vaccine candidate was able to (cross-)neutralize several tested homo- and heterologous ßHPV types *in vitro* and reduced infection by 3 tested ßHPV types *in vivo*, indicating the feasibility of L2-based vaccines against cutaneous ßHPV infection.

To more directly target cutaneous ßHPV types implicated in NMSC development in immunosuppressed patients like organ transplant recipients, we attempted to identify novel ßHPV L2 cross-neutralization epitopes. For the plethora of existing βHPV types broad cross-neutralization and -protection is of paramount importance, since no particular type predominates in NMSC development. Based on a strategy we had successfully developed for the generation of antibodies cross-reactive to dyes used in multi-coloured molecular weight markers [38], we used an innovative sequential immunization regime with N-terminal L2 fragments of several βHPV to generate cross-reactive mAbs. Similar immunization approaches have also been reported for HIV-1 and influenza virus [39–41]. Indeed, four mAb directed against L2 of βHPV types were isolated that showed *in vitro* cross-neutralization as well as *in vivo* cross-protection against a multitude of tested βHPV types. For three of the four mAbs, identified epitopes mapped partially to regions known to contain cross-neutralization epitopes in HPV16 L2, although the epitopes of mAbs 7G9 and 7F6 extend C-terminally. In addition, the 7F6 epitope sequence is highly divergent from HPV16 L2 [26] (suppl. Figure 1) suggesting structural conservation across HPV species. Notably, all of the epitopes were linear, although this is biased by the source of the vaccine antigen and the use of peptides for epitope screening. MAb 7C1 recognizes a minimal epitope homologous to the RG1 epitope [24], and mAb 7G9 is 62.5% homologous to HPV16 L2 aa 71–78, partly overlapping the HVP16 L2 aa 56–75 epitope [25, 42]. MAb 6B11 recognizes an epitope that to our knowledge has no cross-neutralization homolog in HPV16, although the N-terminal half of the 6B11 epitope is homologous to an epitope of a non-neutralizing antibody recognizing HPV16 L2 aa 89–100 [24]. The C-terminal half of the 6B11 epitope, however, is unique to βHPV types because of a 14 aa insertion at this position.

Mice giving rise to the four mAbs 7C1, 7G9, 6B11 and 7F6 were immunized with several (up to five) ßHPV L2 proteins (aa 10–142) in different sequential orders (suppl. table 1), with the rational to empirically promote a cross-neutralizing immune response. However, the order or number of HPV types applied did not impact the cross-neutralization spectrum, nor did the degree of epitope sequence conservation. For example, the mouse that gave rise to mAb 7C1 had been injected with four ßHPV L2 proteins, namely types 8-38-5-20, all of them possessing an identical 7C1 epitope sequence, CPPDVIN (suppl. table 8). Thus, not surprisingly, 7C1 cross-neutralized those ßHPVs with identical epitope sequences such as HPV24 and 96 and in addition the lesser conserved HPV92 with a valine instead of isoleucine at position 6 of the 7C1 epitope. However, a change to leucine at this position was not tolerated, as 7C1 did not cross-neutralize HPV76 (Table 1). From this we predict that HPV types, such as 15 and 23, with an 7C1 epitope identical to HPV76 will not be cross-neutralized by 7C1. Thus, while the 7C1 epitope sequence is highly conserved between ßHPVs, and in general advantageous for mounting cross-reactive immune responses, single aa differences might impair the generation of broadly cross-reactive immune responses indicated by the 7C1 sensitivity to epitope variants. For the chimeric VLP design we extended the 7C1 epitope C-terminally for 3 absolutely conserved aa, KVE, and were indeed able to induce a cross-reactive immune response also towards HPV76. Similar to mAb 7C1, 7G9 was sensitive to single aa changes in its GYVPLGEG epitope sequence and only tolerated a conservative glutamate to glutamine change at position 7 but not changes at other epitope positions present in HPV8, 92 and 96 (suppl. table 8). For the chimeric VLP design we extended the 7G9 epitope N-terminally for 8 highly conserved aa and deleted 2 non-conserved aa form its C-terminus. Immunization with the modified 7G9 epitope VLPs again broadened the cross-reactive response to all tested ßHPVs including HPV8, 92 and 96. The mouse producing mAb 7F6 was immunized with all five beta L2 proteins, including HPV76 L2, but the mAb was not able to neutralize HPV76. The cross-reactivity spectrum of 7F6 revealed that it tolerates variability in the N-terminal, less conserved 4 aa sequence of its 14 aa epitope (Table 1, suppl. table 8 and suppl. Figure 1C). However, deletion of the first aa of this epitope, which is absent from L2 of HPV76 and 96, abolished cross-neutralization by 7F6. Thus, for chimeric VLP design we deleted the N-terminal 4 aa and extended the epitope C-terminally for 2 aa, AE, that are absolutely conserved across ßHPVs (suppl. table 6). Again, these modifications of the epitope resulted in a broadening of the cross-neutralization immune response that now included HPV76 and 96. The most diverse epitope is the 11 aa 6B11 epitope, in which only 3 positions, arginine at position 4, proline at 5 and proline at 9 are absolutely conserved between ßHPVs (suppl. Figure 1C). MAb 6B11 cross-neutralized all 8 HPV types despite the substantial epitope variability but titers were low for HPV5, 20, 76 and 96 *in vitro* and 6B11 failed to significantly protect against HPV5 *in vivo* (Table 1, Fig. 2 and suppl. Figure 2). Extension of the 6B11 epitope for one conserved N-terminal and 3 conserved C-terminal residues in the chimeric VLPs (16L1–6B11short) improved the cross-neutralization response only towards HPV5 but not 76 and 96 and abolished even the ability to neutralize HPV38 (Table 2). Extending the epitope N-terminally for another 5 aa, of which 3 are absolutely conserved (16L1–6B11middle), improved very much the cross-neutralization titers and broadened the spectrum of HPV types neutralized with the exception of HPV76 that possesses the highest sequence variability in the core 6B11 epitope (suppl. Figure 1C). The mAbs 1A1 and 3C6 that were generated from 16L1–6B11middle chimeric VLP immunized mice showed high HPV5 *in vitro* titers (102.400 and 12.800, respectively) and were able to provide HPV5 protection, indicating that the 6B11 epitope does have the capability to mount a protective antibody response to this oncogenic βHPV type (Table 1 and Fig. 2D). We note that, because of the sequential vaccination with epitopes of different HPV types, it is not clear that the HPV5 sequence used in mapping is the optimal one recognized by the MAbs.

Since 7G9 had the highest cross-neutralization titers towards HPV76 but not the broad cross-neutralization spectrum of 6B11, we combined these two epitopes in the 6B11long chimeric VLPs. Insertion of (multiple) repeats of a single L2 cross-neutralization epitope into immunogenic L1-VLP is size limited, at least at the DE-loop position used in this work. Previously, insertion of a maximum of 40 aa resulted in a fusion protein capable to assemble into complete VLP [23]. Herein, insertion of the 6B11long epitope with 41aa length including the GS linker resulted in VLP formation. Although expression of the fusion protein was less efficient and fewer particles were seen by TEM, the cross-neutralizing antibody response both in mice and rabbits was broad covering all tested ßHPVs including HPV76 although at low titers (Table 2 and 3). The less efficient VLP assembly might be the cause for the low titers because even the titer against the HPV16 L1 scaffold of the VLP was substantially reduced compared to the other chimeric VLPs. The less efficient VLP assembly might be balanced by combining two chimeric VLP vaccine candidates, for example with HPV16L1–6B11long providing protection to the group of ßHPV and the addition of the original RG1-VLP that targets all clinically relevant hr and large number of intermediate and lr HPV types [22]. In addition, flanking L2 epitopes by flexible GSGS linkers greatly improved their cross-reactive potential. All chimeric VLP presenting novel ßHPV L2 epitopes via GS linkers were able to mount a broadly neutralizing antibody response in contrast to epitopes that were cloned without linkers into the HPV16 L1 loop (data not shown). Nevertheless, immunizations in mice and rabbits have still highlighted the importance of using a potent adjuvant system to achieve an effective L2-directed response.

Taken together this work identified - by a sequential immunization strategy - cross-neutralization epitopes of cutaneous ßHPV types, of which one is novel and three are conserved across various HPV genera. We show that the HPV16 L1 platform can be successfully employed to accommodate diverse L2 epitopes with different lengths (among other antigens) when inserted into the DE loop. Generation of chimeric L1-L2 VLPs represents an efficient method to produce cross-common experimental vaccines that can protect against the plethora of relevant cutaneous HPV types. Importantly, such an L1-VLP platform is not restricted to HPV epitopes but can easily be adapted to present antigens of unrelated pathogens, particularly if linear protective epitopes are identified, and HPV L1-based chimeric VLPs may therefore open up new avenues for vaccine design against a wide variety of immune subdominant targets.

Additionally, due to their safety, such vaccine candidate as investigated in this work would provide a tremendous advantage for immunosuppressed individuals at great risk to develop skin cancer.

## Supplementary Material

Supplementary Files

This is a list of supplementary files associated with this preprint. Click to download.

- Supplements.pdf

## Figures and Tables

**Table 1:** Cross-neutralization spectra of ßHPV L2 mAbs. Serial dilutions of mAbs were tested for their cross-neutralization against indicated ßHPV types by FC-PBNA, and for animal papillomaviruses MnPV and MmuPV-1 by L2-PBNA (the latter not tested further than 1:100 or 1:200 dilutions, respectively). For types indicated with (#) L2 proteins were not used for immunizations indicative of true cross-neutralization. Neutralization titers are derived from reciprocal serum dilutions resulting in 50% reduction in reporter signal compared to an unrelated mAb or PsV only. Titers of < 50 were considered non-neutralizing (−). n.d., not determined.

	raised against HPV5/8/20/38/76 L2 aa10-141/142				raised against (16L1-) 6B11middle	
	7C1	7G9	6B11	7F6	1A1	3C6
HPV5	16.000	32.000	100	4.000	102.400	12.800
HPV8	8.000	-	1.000	4.000	204.800	25.600
HPV20	32.000	64.000	100	≥ 128.000	204.800	102.400
HPV24^#^	32.000	32.000	32.000	64.000	25.600	3.200
HPV38	8.000	64.000	2.000	128.000	-	400
HPV76	-	16.000	100	-	-	-
HPV92^#^	8.000	-	8.000	≥ 8.000	≥ 409.600	51.200
HPV96^#^	8.000	-	100	-	-	1.600
MmuPV-1^#^	≥ 100	-	-	-	n.d.	n.d.
MnPV^#^	≥ 200	-	-	-	n.d.	n.d.

**Table 2:** Mouse immunizations: Immune sera raised against indicated chimeric fusion proteins/VLP (with 5L2 aa inserts indicated) were pooled per group, serially diluted and tested for (cross-)neutralization against HPV16 by L1-PBNA (*), or indicated ßHPV types by FC-PBNA. Neutralization titers are indicated for reciprocal serum dilutions resulting in 50% reduction in reporter signal compared to pre-serum. Titers of < 50 were considered non-neutralizing and indicated as (−). To analyze immunogenicity in another species, chimeric VLP (except HPV16L1–6B11middle) were adjuvanted with aluminum hydroxide (2% Alhydrogel; Invivogen) and used to immunize one NZW rabbit per antigen (Charles River, France). All immune sera showed antibody titers against HPV16 L1-VLP (ranging from 3.200–12.800) and the respective cognate HPV5 L2 peptides by ELISA (ranging from 200 − 12.800; suppl. table 7). Additionally, all immune sera were able to neutralize HPV16 by L1-PBNA (titers ranging from 200 − 12.800; Table 3), and antisera to 16L1–7G9mod, 16L1–6B11short, 16L1–7F6mod and 16L1–6B11long – but not 16L1–7C1mod – cross-neutralized 5/8, 3/8, 5/8 and 7/8 tested beta HPV types, respectively, by FC-PBNA (Table 3).

HPV		a16L1-7C1mod	a16L1-7G9mod	a16L1-6B11short	a16L1-6B11long	a16L1-7F6mod
5L2 aa #		24–34	60–72	84–98	66–98	127–138
	16*	51.200	51.200	51.200	3.200	12.800
FC-PBNA	5	100	100	800	200	200
	8	200	100	100	100	100
	20	200	50	200	800	≥ 3.200
	24	200	100	200	100	800
	38	100	100	-	100	100
	76	50	100	-	50	100
	92	100	200	50	800	200
	96	200	100	-	50	100

**Table 3:** Serial dilutions of NZW immune sera raised against indicated alum-adjuvanted chimeric VLP were tested for (cross-)neutralization against mucosal hr HPV16 by L1-PBNA, or 8 different beta HPV types by L2-based FC-PBNA, respectively. Neutralization titers are indicated for reciprocal serum dilutions resulting in 50% reduction in reporter signal compared to pre-serum. Titers of < 50 were considered non-neutralizing and indicated as (−).

	HPV	aL1-7C1mod	aL1-7G9mod	aL1-6B11short	aL1-6B11long	aL1-7F6mod
	16	3.200	3.200	12.800	200	12.800
FC-PBNA	5	-	400	-	200	-
	8	-	-	200	400	≥ 800
	20	-	50	-	50	200
	24	-	-	-	400	200
	38	-	200	100	100	-
	76	-	-	-	-	-
	92	-	400	50	200	100
	96	-	200	-	200	400

## Data Availability

The raw data supporting the conclusions of this article will be made available by the authors on request.
